# Capitate sliding osteotomy combined with lunocapitate fusion for stage II/III scaphoid non-union advanced collapse

**DOI:** 10.3389/fsurg.2026.1819731

**Published:** 2026-05-29

**Authors:** Shuang Yang, Yu Si, Fei Xiang, Can Ming Zhong, Ainizier Yalikun, Yue Qi Zhang, Li Li

**Affiliations:** 1 Traditional Chinese Medicine Hospital of Xinjiang Uyghur Autonomous Region, Ürümqi, China; 2 The Second People's Hospital of Kashgar, Kashgar Prefecture, China

**Keywords:** capitate sliding osteotomy, lunocapitate fusion, rigid fixation, stage II/III SNAC, wrist function

## Abstract

**Background:**

Although lunocapitate fusion is currently the most prevalent and effective surgical approach for stage II/III scaphoid non-union advanced collapse (SNAC), clinical challenges such as suboptimal fusion rates and compromised wrist functionality remain unresolved. This study aims to investigate the application of capitate sliding osteotomy combined with central and eccentric fixation techniques in lunocapitate fusion, with the dual objectives of enhancing fusion rates and enabling rigid fixation to facilitate early postoperative rehabilitation, thereby improving postoperative functional outcomes.

**Methods:**

We retrospectively analyzed patients who underwent lunocapitate fusion combined with capitate sliding osteotomy (treatment group) and those who underwent lunocapitate fusion alone (control group) between 2016 and 2024. The average follow-up period was approximately 24 months. These assessments were conducted systematically using various measures, including the Disabilities of the Arm, Shoulder, and Hand (DASH) score, wrist flexion–extension range of motion (ROM), visual analogue scale (VAS) score for pain, and grip strength measurements, preoperatively and at postoperative follow-up assessments.

**Results:**

The treatment group demonstrated significantly superior outcomes compared with the control group at final follow-up. DASH scores improved from 53.91 ± 5.75 preoperatively to 10.73 ± 5.52 postoperatively in the treatment group vs. 51.57 ± 3.94–20.05 ± 3.61 in the control group (*p* < 0.001). VAS pain scores decreased from 5.72 ± 0.87 to 0.71 ± 0.22 in the treatment group vs. 5.38 ± 0.70–1.23 ± 0.25 in the control group (*p* < 0.001). Wrist flexion–extension ROM improved from 80.49° ± 3.36° to 113.74° ± 3.76° in the treatment group vs. 82.54° ± 3.85°–107.25° ± 4.80° in the control group (*p* < 0.001). Grip strength increased from 12.24 ± 1.47 kg to 29.27 ± 2.32 kg in the treatment group vs. 13.53 ± 2.94 kg to 25.56 ± 2.32 kg in the control group (*p* < 0.001). All patients in the treatment group achieved solid fusion.

**Conclusion:**

In this retrospective comparative study, the combination of capitate sliding osteotomy with lunocapitate fusion was associated with higher fusion rates and greater improvements in short-term functional outcomes compared with lunocapitate fusion alone for stage II/III SNAC. This technique appears to be a promising option that may allow for earlier rehabilitation.

## Introduction

Scaphoid fractures are the most prevalent carpal injuries, accounting for 60%–70% of all wrist fractures and primarily affecting young adults (1). Patients with scaphoid fractures may not receive timely treatment because of missed or incorrect diagnoses, potentially resulting in non-union or non-ossification of the fractures. This can eventually lead to the development of ossified wrist arthritis, commonly referred to as scaphoid non-union advanced collapse (SNAC). Up to 75% of patients with scaphoid non-union exhibit radiographic degenerative changes within 4 years (2). The classification system depicted by Shah and Stern categorizes SNAC into four progressive stages, ranging from mild to severe (3). For patients in the early stages of SNAC without concurrent osteoarthritis, open reduction, bone grafting, and refixation are recommended. However, for stage II/III SNAC cases involving multiple joints, the combination of open reduction and bone grafting with scaphoid refixation is insufficient, as this approach does not address the underlying issues of wrist and radiocarpal arthritis.

Current surgical options for stage II/III SNAC include four-corner fusion or lunocapitate fusion, both of which demonstrate comparable fusion rates and complication profiles (4–6). However, approximately 10% of patients undergoing four-corner fusion show delayed union or experience persistent pain (7, 8), partially requiring two surgical procedures (such as total wrist arthrodesis and wrist arthroplasty) (9). In contrast, lunocapitate fusion offers distinct advantages such as a simplified surgical technique, reduced intraoperative trauma, less bone-grafting requirement, superior functional recovery, and a lower incidence of postoperative ulnar-sided wrist pain (10, 11). Duraku et al. demonstrated significantly better wrist flexion at the 12-month follow-up in patients who underwent lunocapitate fusion in comparison with those who underwent four-corner fusion (12). These evidence-based benefits have established lunocapitate fusion as an increasingly recognized treatment for patients with stage II/III SNAC.

With subsequent advancements, the non-union rate of lunocapitate fusion has decreased from an initial average of 33% to the current average of 3.8%–18% (12, 13). However, problems such as poor bone fusion and poor wrist joint function remain unresolved. Therefore, our study attempted the following improvements in the surgical approach to address these issues: (1) We used a combination of cannulated screws and dorsal plates for fixation, achieving both central and eccentric fixation from a biomechanical perspective, increasing initial stability, allowing patients to perform early functional exercises, and maximizing the improvement in wrist joint function. (2) On this basis, we added autologous bone graft and sliding of the capitate, spanning the capitate and lunate bones, to connect the two active bone areas and increase the probability of fusion. Between 2016 and 2024, we treated 50 patients by performing lunocapitate fusion combined with capitate sliding osteotomy. All patients achieved primary fusion of the capitate and lunate bones after surgery, with significant improvement in wrist joint function, pain relief, and good clinical outcomes. We therefore hypothesized that capitate sliding osteotomy combined with lunocapitate fusion would result in higher fusion rates and greater functional improvement compared with traditional lunocapitate fusion alone.

## Materials and methods

**Figure 1:**
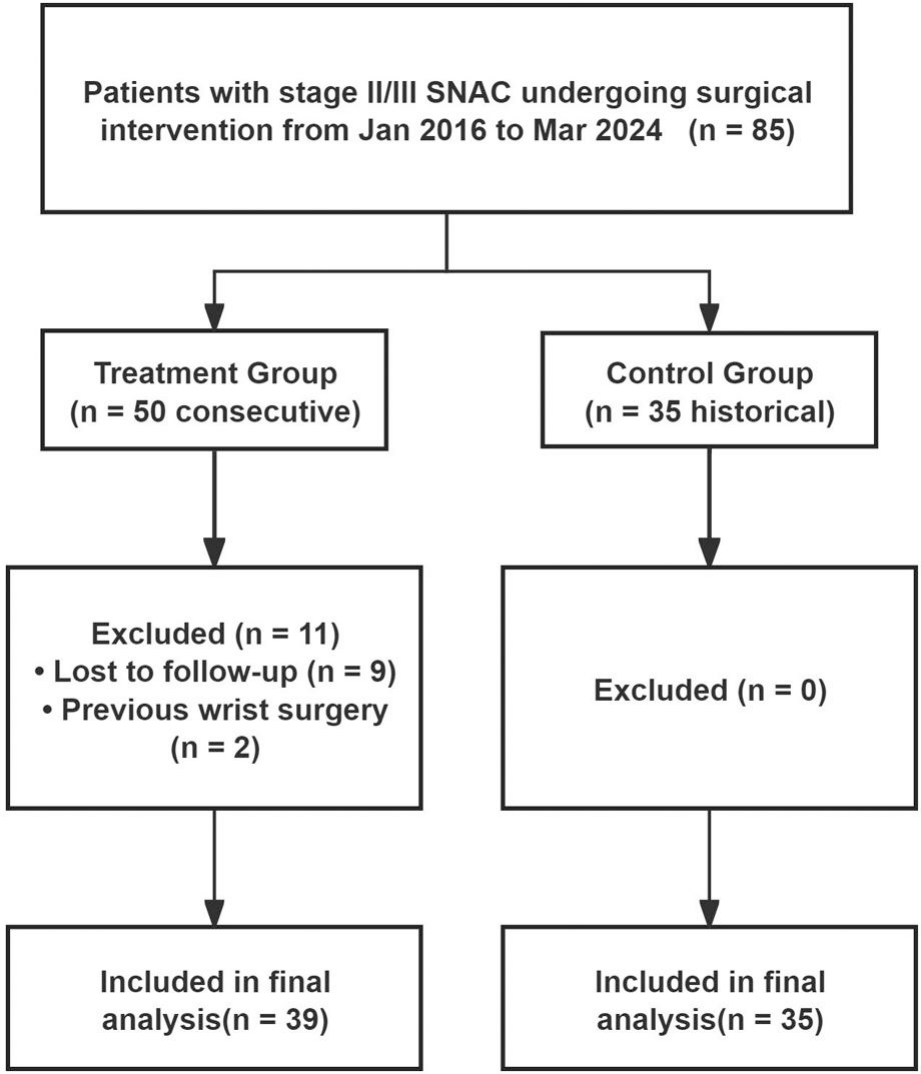
Flowchart illustrating patient selection for a study on stage II or III SNAC with surgical intervention from January 2016 to March 2024, dividing eighty-five patients into a treatment group of fifty and a control group of thirty-five. Eleven patients in the treatment group were excluded, resulting in thirty-nine analyzed, while all thirty-five in the control group were included in final analysis.

Between January 2016 and March 2024, a total of 85 patients with stage II/III SNAC underwent surgical intervention at our institution. Of these, 50 consecutive patients received the novel technique of LC fusion with Capitate Sliding Osteotomy (treatment group), and 35 patients had previously undergone standard LC fusion alone (historical control group). After applying the exclusion criteria, 9 patients were lost to follow-up and 2 had prior wrist surgery, leaving 39 patients in the treatment group for final analysis. The control group consisted of all 35 eligible patients from the historical cohort. A CONSORT-style flow diagram illustrating patient selection is presented in Figure 1. This study was approved by the Institutional Review Board (approval number: 2025XE-GS166). Written informed consent was obtained from all participants or their legal representatives, along with HIPAA authorization for any identifiable health information. The average follow-up period was approximately 2 years. Participants were excluded if they had the following: (1) history of previous wrist surgery, (2) concurrent scapholunate advanced collapse (SLAC) conditions, (3) other carpal instability patterns requiring different surgical interventions, (4) active infection or malignancy, and (5) severe medical comorbidities precluding surgery.

Functional outcomes were systematically assessed using the Disabilities of the Arm, Shoulder, and Hand (DASH) score, wrist flexion–extension range of motion (ROM), visual analogue scale (VAS) for pain, and grip strength measurements. The patients completed standardized evaluations preoperatively and every 6 months postoperatively. The validated DASH questionnaire, which consists of 30 items evaluating pain and disability during the preceding week, was administered at each time point. Average pain intensity was measured using a 10-cm VAS, anchored at 0 (“no pain”) and 10 (“worst imaginable pain”). For wrist ROM measurements, certified hand therapists measured active flexion–extension arcs using a standardized goniometric protocol (mean of three trials). Grip strength measurements were recorded at the second handle position with the elbow flexed at 90° and forearm neutral. The average of three maximal voluntary contractions was calculated.

### Statistical analysis

We first conducted a rigorous analysis of the collected data. Normality tests were used to verify that the data conformed to a normal distribution, and the results were presented in the form of mean ± standard deviation. Meanwhile, paired t-tests were employed to compare the differences in measurements between the preoperative period and the last follow-up. This test method is suitable for comparing the measurements of the same research subject at different time points and can effectively evaluate the effect of the intervention measures.

Primary Outcome: The primary outcome was radiographic fusion of the lunocapitate joint. Fusion was assessed by two independent, blinded reviewers (a hand surgeon and a musculoskeletal radiologist) using standard posteroanterior and lateral radiographs at 2, 3, 6, and 12 months postoperatively. Solid fusion was defined as the presence of bridging trabeculae crossing at least 50% of the lunocapitate interface on both radiographic views, accompanied by the absence of any radiolucency around the screws, loss of implant position, or hardware failure.

Secondary Outcomes: (1) DASH score: Validated 30-item questionnaire assessing upper extremity disability (0 = no disability, 100 = maximal dysfunction). (2) VAS for pain: 10-cm scale anchored at 0 = “no pain” and 10 = “worst imaginable pain”. (3) Wrist ROM: Active flexion–extension arc measured with standardized goniometry. (4) Grip strength: Measured using a Jamar dynamometer (average of three trials).

### Surgical techniques

**Figure 2:**
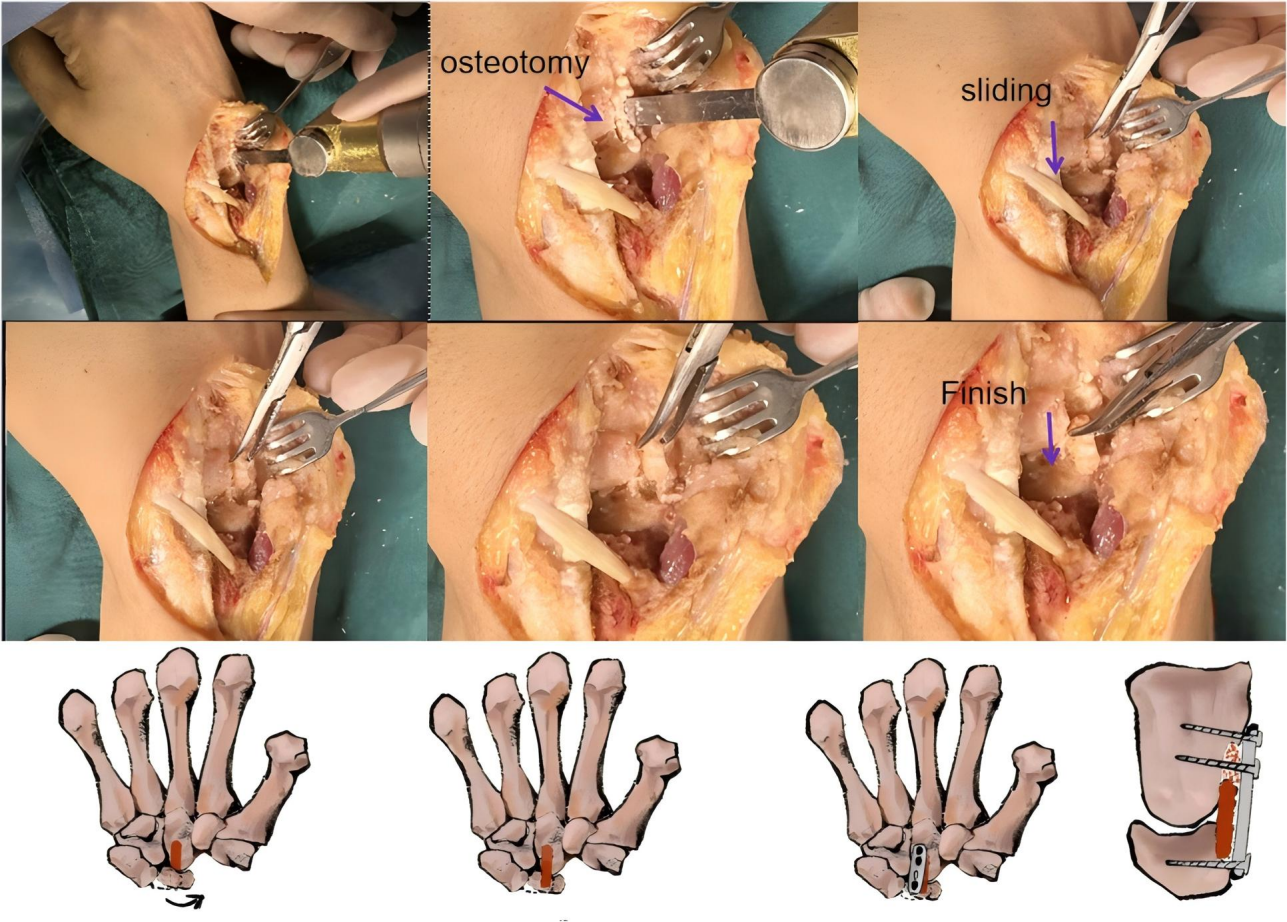
Surgical illustration of the capitate sliding osteotomy combined with lunocapitate fusion. Exposure of the midcarpal joint and lunocapitate articulation; osteotomy of the capitate and harvesting of a bone block ;sliding the bone block to bridge the lunocapitate joint; fixation with two hollow screws (central fixation) and a dorsal metacarpal plate (eccentric fixation).

All surgeries (both control and treatment groups) were performed by two highly qualified senior surgeons. After induction of neuraxial anesthesia at the brachial plexus, the patient was placed in the supine position. Subsequently, standard skin antisepsis was performed, and sterile drapes were placed. Tourniquets were applied to the patient's upper arm to maintain a pressure of 35 kPa. By creating an S-shaped incision approximately 6 cm in length on the dorsal side centered on the lunocapitate joint, the skin and subcutaneous tissues were incised progressively. The extensor tendons were then pulled aside, and the fascia layer and joint capsule were cut to expose the midcarpal joint and confirm the lunocapitate joint. First, the non-union site of the scaphoid fracture was exposed and local ossification was identified. A portion of the scaphoid bone was either partially or completely excised. Subsequently, osteotomy was performed obliquely at the site of impact between the radial styloid process and the scaphoid while ensuring no impact when the wrist joint was excessively radialized. We rotated the lunate bone radially to achieve a better congruence with the lunocapitate joint surface. After confirming the lunocapitate joint surface, the articular cartilage was removed, and trial reduction was attempted. A guidewire was then used for stabilization. Once joint alignment was accurately confirmed using C-arm fluoroscopy, two hollow screws were fixed in place. In the treatment group, an accurately cut bone block measuring approximately 10 mm  × 2 mm × 2 mm was harvested from the capitate bone surface, and a matching depression of the same shape was created at the corresponding location on the lunate bone. The bone block was slid from the capitate bone to the lunocapitate joint and inserted into the depression; the cancellous bone harvested from the scaphoid was placed into the groove of the capitate and the lunocapitate joint and fixed in place using a 1.5 metacarpal plate. After confirming correct positioning through C-arm fluoroscopy, the incision was closed layer by layer. After central screw fixation, the fusion site was augmented with cancellous bone graft obtained from the excised scaphoid. No capitate osteotomy, sliding bone block, or dorsal plating was performed. The procedure concluded after definitive screw fixation and bone grafting. The surgical illustration is shown in Figure 2.

### Postoperative management

**Figure 3:**
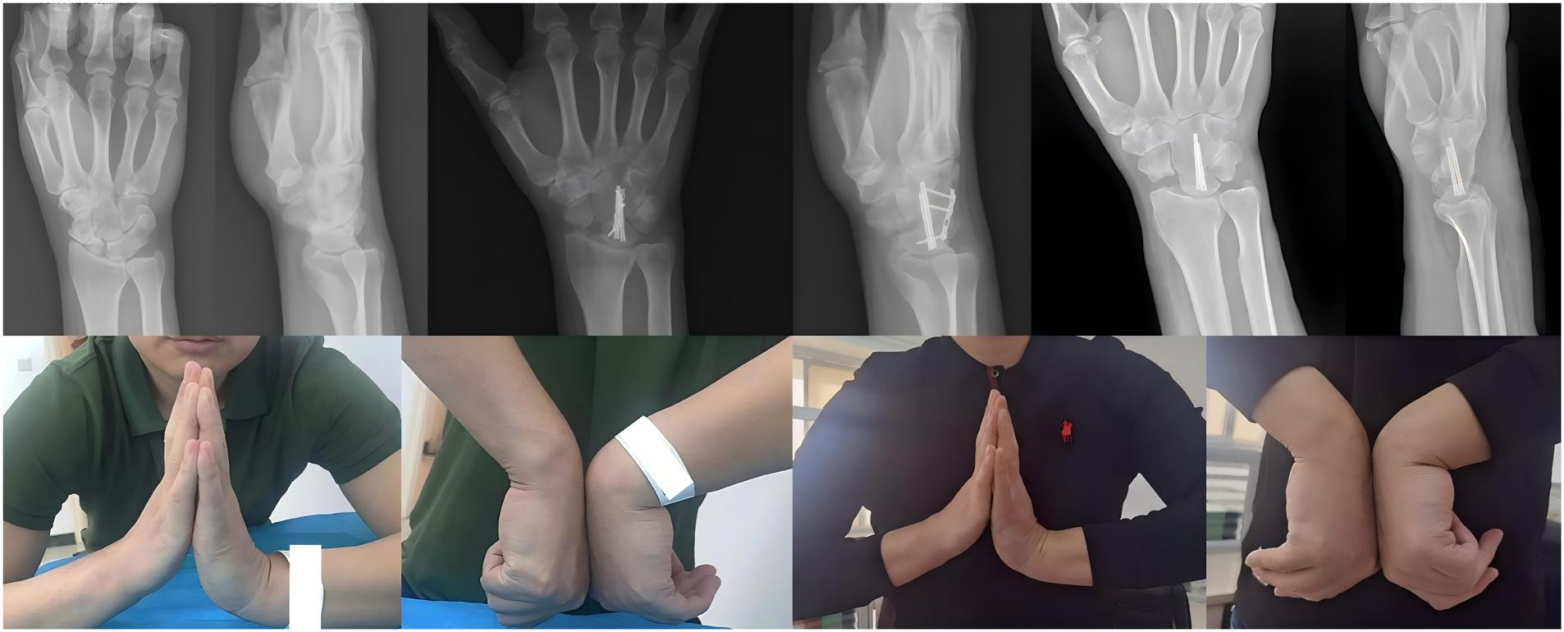
A typical case example. Preoperative and postoperative radiographs and clinical photographs.

Postoperatively, the patient was placed in a functional cast for 2 weeks to maintain stability at the surgical site and promote tissue healing. On the second day after surgery, the patient began finger flexion and extension exercises while wearing the cast, with each session lasting 15–20 min and performed 3–4 times daily to enhance joint mobility and muscle strength in the fingers. After 2 weeks, the cast and sutures were removed, followed by the use of a temporary brace for support and protection. During the rehabilitation period from the second to fourth week postoperatively, the intensity and frequency of finger functional exercises were gradually increased, with each session extending to 20–30 min and performed 4–5 times daily to further improve flexibility and strength. After 4 weeks, the temporary brace was removed, and under the guidance of a professional therapist, the patient began to perform wrist joint functional exercises, including active movements and low-load passive movements, each lasting 10–15 min and performed 3–4 times daily to promote mobility and coordination of the wrist joint. Throughout the entire rehabilitation process, therapists developed personalized rehabilitation plans based on the patient's specific conditions and recovery progress, providing close supervision and guidance to ensure the safety and effectiveness of the rehabilitation training, helping the patients recover hand and wrist function as soon as possible, and improving the patients' quality of life. The postoperative protocols were completely consistent between the two groups. A typical case is shown in Figure 3.

## Results

The groups were comparable in terms of age, gender distribution, affected side, and SNAC stage (Table 1).

**Table 1:** Demographic and baseline characteristics.

Characteristic	Treatment group (*n* = 35)	Control group (*n* = 39)	*P*
Age (years)	32.8 ± 5.4	31.9 ± 5.6	0.69
Gender (male/female)	26/9	32/7	0.36
Affected side (right/left)	31/4	30/9	0.22
Follow-up (months)	25.4 ± 3.2	25.8 ± 3.3	0.62

### Primary outcome: fusion rates

All patients in the treatment group (39/39, 100%) achieved solid fusion compared with 30/35 (85.7%) in the control group (*p* = 0.026). Time to fusion was significantly shorter in the treatment group (10.2 ± 2.1 weeks vs. 14.6 ± 3.4 weeks, *p* = 0.001).

### Secondary outcomes

**Figure 4:**
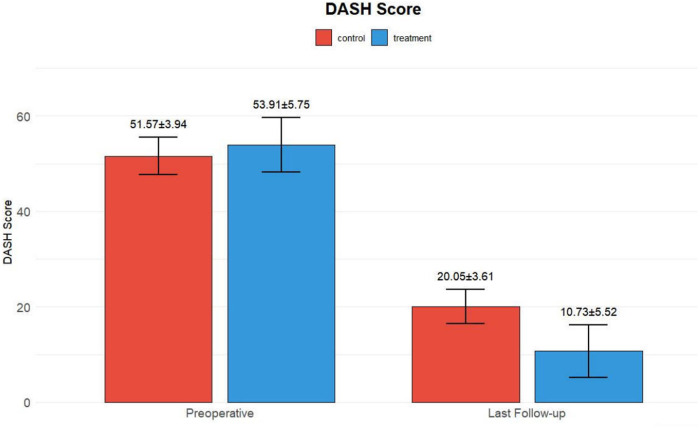
Comparison of DASH scores between two groups before surgery and at final follow-up. Scores decreased more obviously in the treatment group (*p* < 0.001).

**Figure 5:**
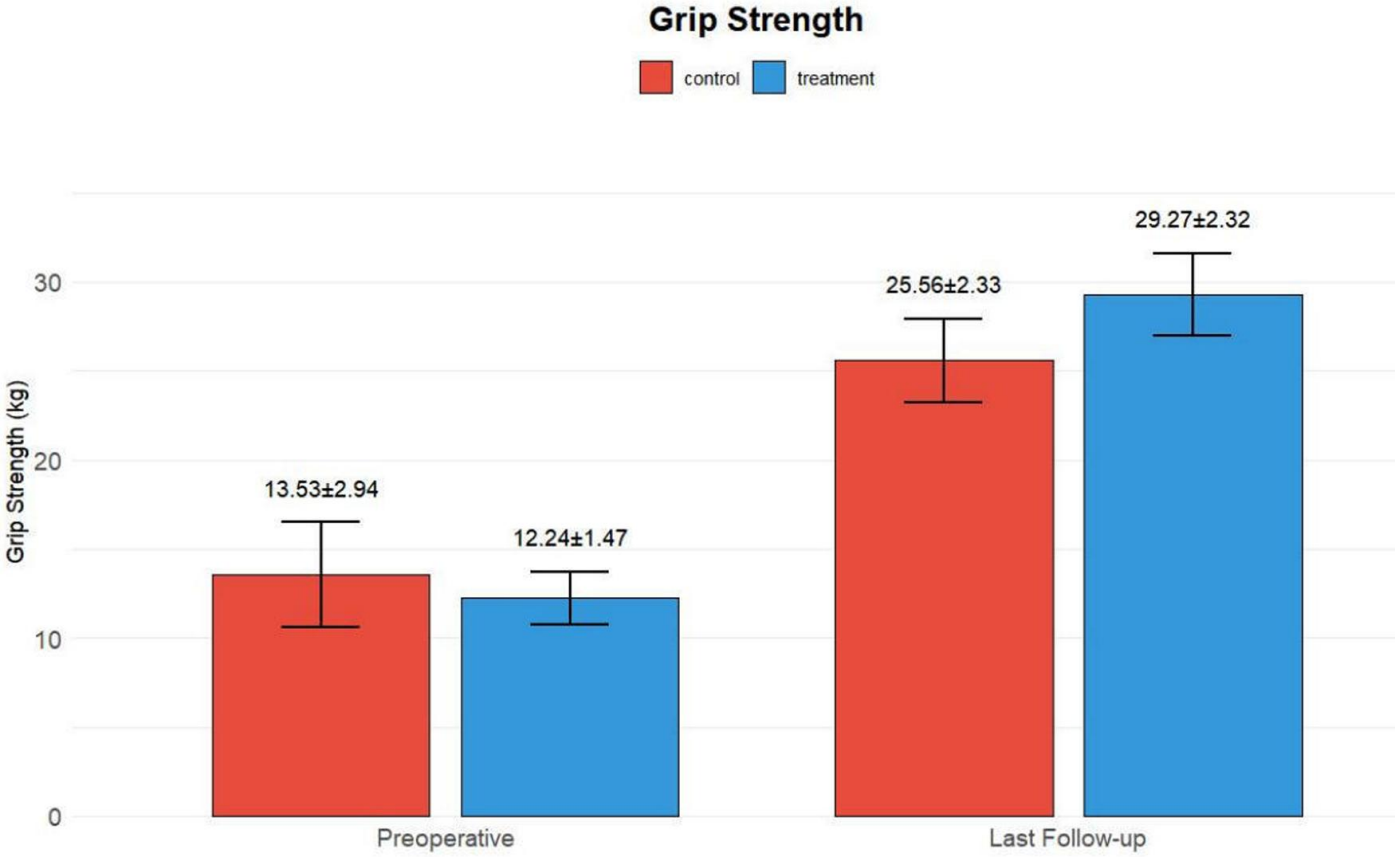
Comparison of grip strength between two groups before surgery and at final follow-up. Strength improved more obviously in the treatment group (*p* < 0.001).

**Figure 6:**
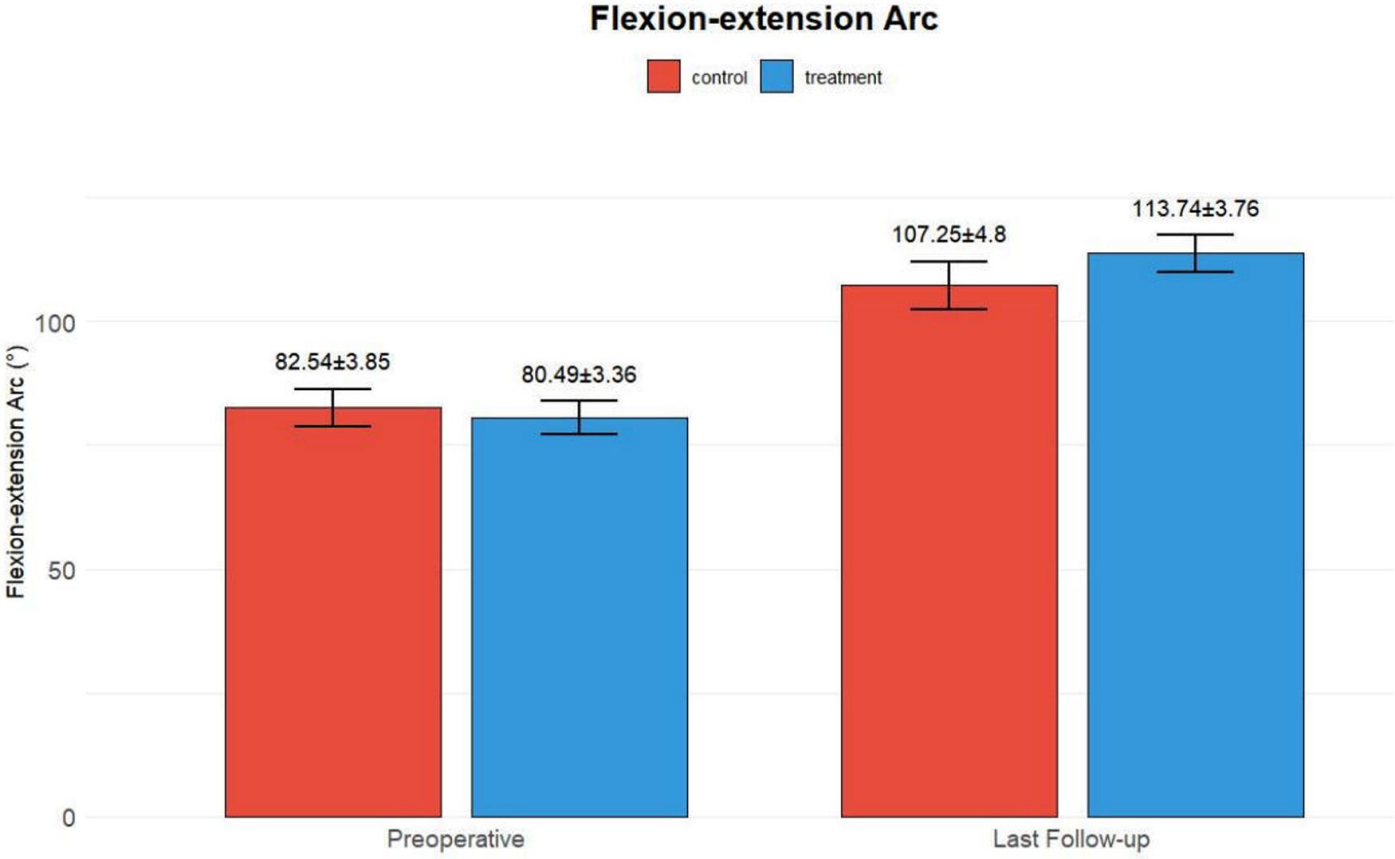
Comparison of wrist flexion-extension range between two groups before surgery and at final follow-up. Mobility improved more obviously in the treatment group (*p* < 0.001).

**Figure 7:**
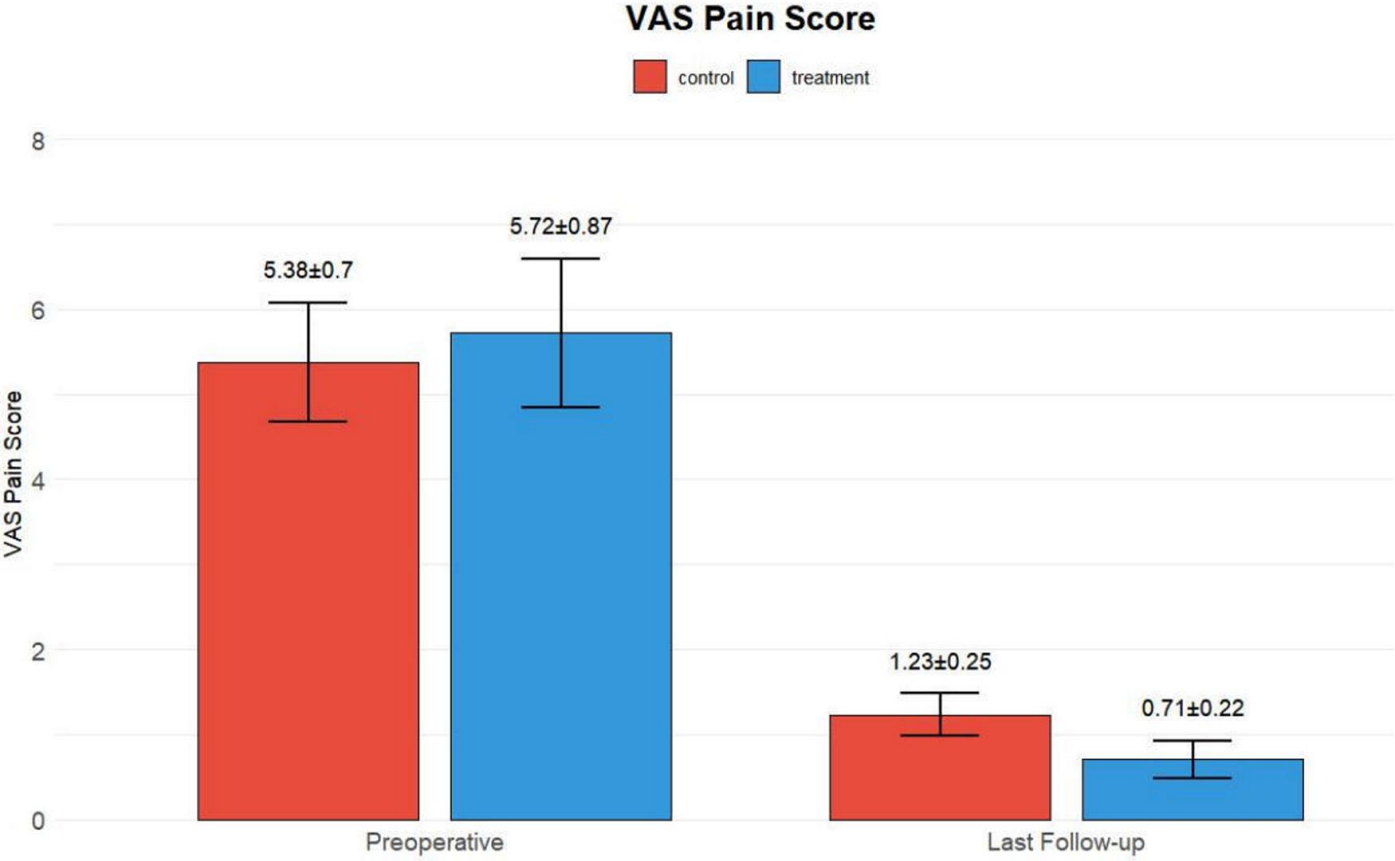
Comparison of VAS pain scores between two groups before surgery and at final follow-up. Pain relief was more significant in the treatment group (*p* < 0.001).

The treatment group demonstrated significantly superior outcomes compared with the control group at final follow-up. DASH scores improved from 53.91 ± 5.75 preoperatively to 10.73 ± 5.52 postoperatively in the treatment group vs. 51.57 ± 3.94–20.05 ± 3.61 in the control group (*p* < 0.001). VAS pain scores decreased from 5.72 ± 0.87 to 0.71 ± 0.22 in the treatment group vs. 5.38 ± 0.70–1.23 ± 0.25 in the control group (*p* < 0.001). Wrist flexion–extension ROM improved from 80.49°±3.36° to 113.74° ± 3.76° in the treatment group vs. 82.54° ± 3.85° to 107.25° ± 4.80° in the control group (*p* < 0.001). Grip strength increased from 12.24 ± 1.47 kg to 29.27 ± 2.32 kg in the treatment group vs. 13.53 ± 2.94 kg to 25.56 ± 2.32 kg in the control group (*p* < 0.001). In addition, we performed a between-group comparison of change scores (improvement from baseline). This approach adjusts for baseline intergroup variability and yields a more reliable assessment of therapeutic effect. For outcome measures of the preoperative period and last follow-up or statistical graphs of the preoperative period and last follow-up result indicators, refer to Tables 2, 3 and Figures 4–7.

**Table 2:** Outcome measures of the preoperative period and last follow-up.

Outcome measure	Preoperative control	Treatment	*P*	Last follow-up control	Treatment	*P*
DASH score	51.57 ± 3.94	53.91 ± 5.75	0.044	20.05 ± 3.61	10.73 ± 5.52	<0.0001
VAS pain score	5.38 ± 0.70	5.72 ± 0.87	0.067	1.23 ± 0.25	0.71 ± 0.22	<0.0001
Flexion–extension arc	82.54 ± 3.85	80.49 ± 3.36	0.018	107.25 ± 4.80	113.74 ± 3.76	<0.0001
Grip strength (kg)	13.53 ± 2.94	12.24 ± 1.47	0.024	25.56 ± 2.33	29.27 ± 2.32	<0.0001

**Table 3:** Comparison of mean change from baseline to final follow-up.

Outcome measure	Treatment group (*n* = 39) Δ ± SD	Control group (*n* = 35) Δ ± SD	Mean difference	T	*P*
Δ DASH score	−43.18 ± 6.67	−31.53 ± 1.95	−11.65	10.43	<0.001
Δ VAS pain score	−5.02 ± 0.84	−4.16 ± 0.68	−0.86	4.94	<0.001
Δ Flexion–extension ROM (°)	+33.26 ± 5.19	+24.71 ± 5.62	8.55	6.75	<0.001
Δ Grip strength (kg)	+17.03 ± 2.19	+12.03 ± 1.28	5	12.13	<0.001

### Between groups

#### Complications

No major complications were observed in either group. Minor complications included: Treatment group: one case of superficial wound infection (resolved with antibiotics). Control group: three cases of delayed union requiring prolonged immobilization, two cases of non-fusion, in which patients achieved healing after undergoing the surgical method of the treatment group.

#### Subgroup analysis

SNAC Stage II vs. III: Both groups demonstrated similar improvements regardless of SNAC stage, with no significant interaction effects between treatment and stage (*p* > 0.05 for all outcomes).

## Discussion

The findings of this study suggest that capitate sliding osteotomy combined with lunocapitate fusion is associated with superior clinical outcomes compared with traditional lunocapitate fusion alone for stage II/III SNAC. The key findings include significantly higher fusion rates (100% vs. 85.7%), improved functional scores, reduced pain, and enhanced range of motion and grip strength.

Watson et al. (14, 15) reported that partial wrist arthrodesis is typically required to achieve satisfactory clinical outcomes in patients with scaphoid non-union complicated by wrist osteoarthritis. Previous studies have also identified complications such as non-union of fracture sites and wrist pain with limited ROM after four-corner fusion and lunocapitate fusion. We have also observed certain adverse outcomes, including compromised consolidation following lunocapitate arthrodesis, inadequate fixation stability, and unsatisfactory postoperative functional recovery. From the perspective of internal fixation techniques, the traditional Kirschner wire and hollow-screw fixation methods have limitations in terms of stability, and the fixation methods are relatively monotonous. In early studies on lunocapitate fusion, the high incidence of non-union was attributed to deficiencies in fixation techniques. During the Kirschner wire application period, the non-union rate reached 50%. However, with the use of hollow screws, some reports (16) suggest that the rate of lunocapitate joint non-union has been reduced to below 10%, but the rates in actual clinical practice are higher. Recent research has indicated that the rate of non-union is significantly equal to or lower than that reported in previous studies on four-corner fusion techniques (8, 13). However, among patients with prolonged non-union, some experienced loosening of fixation or broken screws. As a result, some physicians provide patients with plaster casting external fixation for 6–8 weeks to ensure effective fixation.

This may be attributable to incomplete and insufficient cleaning of the lunocapitate joint surfaces during surgery, which is one of the reasons for poor fusion after lunocapitate fusion surgery. Therefore, during the surgical process, the subchondral bone should be cleaned as much as possible to improve the likelihood of fusion. The cleaning should be sufficiently thorough to visualize punctate bleeding after tourniquet application. If the bleeding is poor, local drilling can be performed using a small drill bit.

To improve the fusion rate of lunocapitate fusion, we proposed a new surgical method that involves capitate sliding osteotomy combined with central and eccentric fixation. To address the shortcomings of previous lunocapitate fusion techniques, we used two hollow screws for parallel or crisscross fixation of the lunocapitate joint, which allowed for either proximal or distal insertion of the screws to achieve a central fixation. However, because of the relatively small patient population, we were not able to conduct an effective comparison; therefore, we could not confirm which method of nail insertion was superior. Each patient's condition was assessed individually during surgery. If a patient's wrist joint could be bent to a great extent toward the palm, exposing more than half of the joint surface of the lunate, we recommended inserting the screws from the palmar side in a straight, forward direction. In contrast, for patients showing restriction in the wrist's ROM, we recommended inserting the screws from the distal end in a retrograde manner. Furthermore, on the dorsal side, a dorsal locking plate was used to fix the metacarpal bones, which served as an eccentric fixator. In combination with central fixation, this approach ensured solid fixation, increasing the stability coefficient of the lunocapitate joint and enhancing the early stability of lunocapitate fusion. This allowed patients to engage in functional exercises earlier, resulting in better postoperative function and effectively promoting fusion of the lunocapitate joint. However, because of the small interosseous space between the radius and the lunate, the dorsal plate may collide with the distal end of the radius in rare cases when the wrist is fully dorsiflexed. If such collisions occurred, part of the distal radius was excised until the collision stopped. The excised portion (which could be a part of the lunate or cancellous bone of the distal radius) can be used to fill the distal defect in the capitate to improve the fusion rate. We performed an osteotomy on the capitate bone and then slid the osteotomy fragment to bridge over the lunocapitate joint onto the lunate to enhance early stability and improve the fusion rate of the dorsal wrist lunocapitate joint. This bone fragment is active and can slide from one cortical bone area to another. This allowed for an increase in the probability of healing between the capitate and lunate bones. We finally decided to harvest a bone graft measuring approximately 10 mm × 2 mm × 2 mm. In the surgical procedure, the size of the harvested bone graft did not affect the structural integrity of the capitate bone and did not compromise the stability of fixation with hollow screws. Second, the sliding bone graft was kept as intact and well fitted as possible. The sliding bone graft was positioned ingeniously across the osteonecrotic area, making it a critical bridge between the two osteoinductive zones and thus enabling the bone-formation channel technique (17, 18).

Various surgical techniques, each with certain limitations that restrict patients' ability to engage in early postoperative physical activity, are available at present. However, the surgical method we employed is expected to overcome these limitations, creating conditions for the patients to begin their recovery and movement sooner.

In the present study, the combination of lunocapitate fusion with sliding osteotomy significantly improved the surgical success rate. The core advantage of this surgical technique is reflected not only in the high postoperative fusion rate but also in its strong fixation design, which allowed patients to start functional activities early and thereby effectively alleviated postoperative wrist pain and promoted recovery of function. The treatment group demonstrated superior outcomes compared with the control group in DASH scores, VAS scores, grip strength, and wrist flexion–extension range of motion at the last follow-up, with a higher rate of excellent and good results. In addition, all treatment group patients in this study achieved a one-stage fusion of the lunocapitate joint postoperatively, and the functional assessments and related scores of all patients demonstrated good outcomes. Notably, the treatment group postoperative recovery time was significantly shorter than that reported in previous studies. Throughout the follow-up period, both clinical examinations and radiological assessments indicated satisfactory surgical results, further validating the feasibility and effectiveness of the surgical technique. These findings suggest potential clinical benefits of this modified technique. However, given the retrospective design, limited follow-up duration, and use of historical controls, these results should be interpreted as preliminary in nature. Although the technique shows promise, further validation through prospective randomized controlled trials is necessary before it can be recommended as a standard treatment option for stage II/III SNAC.

### Limitations

Lunocapitate fusion combined with sliding osteotomy is not suitable for all patients with old scaphoid fractures. This surgical approach is more effective in young and active patients. However, careful consideration is warranted in elderly patients or those with severe osteoporosis because of their diminished healing potential and increased surgical risk. In addition, the nature of the fracture and the blood supply to the fracture site are crucial factors in formulating the surgical strategy. Consequently, in clinical practice, individualized treatment plans should be tailored to the unique circumstances of each patient to optimize the treatment outcomes.

Several important limitations of this study must be acknowledged. First and foremost, this is a retrospective, non-randomized comparative study with a historical control group. This design inherently introduces the potential for selection bias and temporal bias, as patient characteristics, perioperative management, and outcome assessment could have evolved over the study period. Although we applied strict inclusion and exclusion criteria and all surgeries were performed by the same two senior surgeons using standardized postoperative protocols, unmeasured confounders may still exist. Second, the relatively small sample size from a single institution limits the generalizability of our findings and may have inflated the risk of a Type I error. Future prospective, randomized controlled trials with larger, multicenter cohorts are necessary to confirm these preliminary results. Third, the follow-up duration, while averaging 2 years, is considered short term. Longer follow-up is required to assess the durability of the functional outcomes and to detect late complications such as implant failure or secondary osteoarthritis in adjacent joints.

## Data Availability

The original contributions presented in the study are included in the article/Supplementary Material; further inquiries can be directed to the corresponding author.
